# Effect of pupil dilation on biometry measurements and intraocular lens power in eyes with high myopia

**DOI:** 10.3389/fmed.2022.963599

**Published:** 2022-10-19

**Authors:** Wenqun Xi, Mingmin Yang, Jinci Wan, Yuan Wang, Yuanjiao Qiao, Xiaosheng Huang, Xinhua Liu, Ning Fan, Shenwen Liu, Kun Zeng, Sheng Chen

**Affiliations:** Shenzhen Key Laboratory of Ophthalmology, Shenzhen Eye Hospital, Affiliated Hospital of Jinan University, Shenzhen, China

**Keywords:** cataract, pupil dilation, IOLMaster, biometry, IOL power calculation, high myopia

## Abstract

**Purpose:**

The present study sought to evaluate the effects of pupil dilation on ocular parameter measurements and intraocular lens (IOL) power calculation using IOLMaster in highly myopic cataract patients.

**Materials and methods:**

A total of 233 eyes were included in this prospective study and assigned to four groups based on range of axial length (AL) as follows: group A:26–28 mm, group B:28–30 mm, group C:30–32 mm, and group D:32–36 mm. Flattest and steepest keratometry (K1 and K2), AL, anterior chamber depth (ACD), lens thickness (LT), and white-to-white (WtW) were determined using IOLMaster before and after administration of topical tropicamide. The corresponding IOL powers were calculated using Sanders–Retzlaff–Kraff/theoretical (SRK/T), Haigis, and Barrett Universal II formulas.

**Results:**

Variations in AL, K1 and K2 following dilation were not significant (*P* > 0.05 in all groups). The results showed that ACD increased significantly after dilation (*P* = 0.000 in all groups), whereas LT decreased significantly after dilation (*P* = 0.000, 0.000, 0.001, and 0.003). Post-dilation WtW increased significantly in Group A, B, and C (*P* = 0.001, 0.001, and 0.025) but not in Group D. When IOL power was calculated as a discrete variable, significant differences were observed between pre- and post-dilation IOL power.

**Conclusion:**

Pupil dilation in cataract eyes with high myopia does not cause significant changes in AL and K. However, it significantly increases ACD as well as WtW values and significantly decreases the LT value. Surgeons should evaluate the effect of pupil dilation on IOL power prediction as the present findings show extreme cases. Notably, Barrett Universal II formula had the best concordance between different pupil conditions in long eyes.

## Introduction

Recent advances in cataract surgery have increased the safety and efficacy of this common practice, and transformed the goal of cataract surgery from purely visual rehabilitation to a refractive procedure. Precise biometric measurement and proper selection of intraocular lens (IOL) is important for achieving desirable refractive outcomes after cataract surgery (1). Axial length (AL) and corneal power (K) are essential parameters for various IOL power calculation formulas (2, 3). The most widely used third-generation formula is the Sanders–Retzlaff–Kraff/theoretical (SRK/T) formula, which only requires AL and K values. Later devised fourth-generation Haigis formula utilizes AL, K, and anterior chamber depth (ACD) for IOL power calculation. The fifth-generation Barrett Universal II formula uses AL, K, ACD, lens thickness (LT), and white-to-white measurement (WtW) for theoretically more accurate estimation (3).

IOLMaster (Carl Zeiss Meditec) was the first commercially available ocular biometer and is considered as the reference standard tool. Previous studies utilized IOLMaster to evaluate the effects of pupil dilation on biometric measurements and IOL power calculation for eyes of average AL (2, 4–10). Eyes with high myopia (AL ≥ 26.0 mm) present with high risk of early-onset nuclear cataracts and pathologic changes in the choroid and retina. Therefore, routine dilated fundus examinations are recommended for pre-operation cataract patients with high myopia. However, studies have not explored the effects of pupil dilation on biometric measurements for cataract patients with high to extremely high myopia. In the present study, the effects of pupil dilation on ocular parameter measurements in highly myopic cataract patients was explored using IOLMaster 700. Moreover, corresponding IOL power calculation consistency of the SRK/T, Haigis, and Barrett Universal II formula were evaluated.

## Materials and methods

### Subjects and study design

This prospective study was conducted at the cataract pre-operation clinic of Shenzhen Eye Hospital. The study was conducted following the tenets of the Declaration of Helsinki. The study protocol was reviewed and approved by the institutional review board of Shenzhen Eye Hospital. Written informed consents were obtained from all participants prior participation in the study. A total of 233 eyes of 60 men and 81 women were included in this study between June 1, 2021, and January 31, 2022. The mean age of the subjects enrolled in this study was 58.13 ± 11.92 years (range, 29–84 years). The inclusion criteria was cataract patients with high myopia (26.0 mm ≤ AL < 36.0 mm). The exclusion criteria were as follows: mydriasis of less than 6.0 mm, previous ocular surgery or trauma, previous corneal refractive surgery, history of contact lens wear, severe dry eye, corneal opacities, elevated scars, narrow iridocorneal angle, glaucoma, any retinal or choroidal disease (except posterior staphyloma), ocular inflammatory disease, inability to widely open the eyelids, and poor ocular fixation. Patients with physical or mental inability to cooperate with the IOLMaster 700 examination were also excluded.

All participants were subjected to two consecutive ocular biometry measurements; before and after pupil dilation. Briefly, IOLMaster 700 examination was conducted for each patient before pupil dilation according to the manufacturer’s standard instructions. The pupils were then dilated four times, 5 min apart with 0.5% tropicamide 45 min before the subsequent measurement. A second IOLMaster 700 measurement was performed by the same experienced technician specialized for cataract evaluation after mydriasis was attained (confirmed by the disappearance of pupillary light reflex). The IOLMaster 700 measurement data included AL, flattest and steepest K (K1 and K2), ACD, LT, and WtW were recorded. The IOL power required for emmetropia before and after pupil dilation, with a target refraction of −0.5D, was calculated using the IOLMaster 700 device software. The SRK/T, Haigis, and Barrett Universal II formula with the Tecnis monofocal one-piece ZCB00 model (Abbot Medical Optics) with an A constant of 119.3 was used to compute the IOL power.

### Statistical analysis

Statistical analysis was conducted using the Statistical Package for Social Science (SPSS Inc., Chicago, IL, USA) version 22.0. Data were expressed as mean ± standard deviation. Ocular parameter changes before and after pupil dilation were evaluated using the paired *t*-test. *P* < 0.05 was considered statistically significant. Based on previous studies (2, 6), a sample size calculation with α = 0.05 and β = 80% indicates that each group requires a minimum of 36 subjects to give an 80% power at 5% two-sided significance level for the statistical test to detect the difference.

## Results

The study participants were assigned into four groups based on AL for comparisons. Group A (26.0 mm ≤ AL < 28.0 mm) comprised 85 eyes of 28 men and 27 women. Participants in Group A had a mean age of 58.95 ± 11.38 years (range, 35–79 years). Group B (28.0 mm ≤ AL < 30.0 mm) comprised 63 eyes of 11 men and 26 women. Participants in Group B had a mean age of 57.32 ± 12.01 years (range, 29–84 years). Group C (30.0 mm ≤ AL < 32.0 mm) comprised 47 eyes of 13 men and 15 women. The mean age of participants in Group C was 57.14 ± 14.06 years (range, 29–82 years). Group D (32.0 mm ≤ AL < 36.0 mm) comprised 38 eyes of 8 men and 13 women. The mean age of participants in Group D was 58.71 ± 10.65 years (range, 45–81 years). There was no statistically significant difference in age (*P* = 0.933) and sex ratio (*P* = 0.221) among the four groups.

IOLMaster 700 pre- and post-dilation measurement data for all groups included AL, K1, K2, ACD, LT, WtW, and the differences between them are presented in Table 1. The findings showed that changes in AL, K1 and K2 values between pre- and post-dilation in the four groups were not statistically significant (*P* > 0.05). Post-dilation ACD was significantly deeper compared with that before dilation in the four groups (*P* = 0.000 in all groups). Post-dilation LT was significantly thinner relative to that before dilation for all groups (*P* = 0.000, 0.000, 0.001, and 0.003). Significant increase in post-dilation WtW was observed in Group A, B, and C compared with the pre-dilation WtW measurements (*P* = 0.001, 0.001, and 0.025).

**TABLE 1:** The biometric data obtained using IOLMaster 700 before and after pupil dilation.

		Mean ± SD				
				
Parameter	Group	Pre-dilation	Post-dilation	Difference	*t*	*p*
AL (mm)	A	26.94 ± 0.56	26.92 ± 0.56	−0.015 ± 0.079	–1.741	0.085
	B	29.05 ± 0.61	29.06 ± 0.64	0.009 ± 0.1139	0.653	0.516
	C	30.87 ± 0.55	30.86 ± 0.57	−0.011 ± 0.098	–0.741	0.462
	D	33.32 ± 1.13	33.33 ± 1.17	0.005 ± 0.314	0.093	0.926
ACD (mm)	A	3.50 ± 0.33	3.59 ± 0.33	0.090 ± 0.063	13.070	0.000
	B	3.51 ± 0.35	3.60 ± 0.35	0.094 ± 0.068	10.935	0.000
	C	3.47 ± 0.38	3.59 ± 0.42	0.116 ± 0.168	–4.740	0.000
	D	3.30 ± 0.48	3.41 ± 0.52	0.109 ± 0.090	7.480	0.000
LT (mm)	A	4.29 ± 0.42	4.28 ± 0.42	−0.014 ± 0.023	–5.578	0.000
	B	4.28 ± 0.38	4.26 ± 0.39	−0.022 ± 0.037	–4.757	0.000
	C	4.49 ± 0.39	4.48 ± 0.40	−0.015 ± 0.028	3.703	0.001
	D	4.63 ± 0.52	4.61 ± 0.52	−0.018 ± 0.036	–3.196	0.003
WtW (mm)	A	11.98 ± 0.45	12.06 ± 0.49	0.081 ± 0.226	3.312	0.001
	B	11.83 ± 0.43	11.92 ± 0.45	0.090 ± 0.208	3.445	0.001
	C	11.83 ± 0.49	11.89 ± 0.49	0.060 ± 0.177	2.314	0.025
	D	11.77 ± 0.45	11.83 ± 0.44	0.068 ± 0.211	2.002	0.053
K1 (D)	A	42.83 ± 1.24	42.80 ± 1.26	−0.027 ± 0.261	–0.658	0.513
	B	43.09 ± 1.10	43.09 ± 1.23	−0.008 ± 0.485	–1.30	0.897
	C	43.41 ± 1.49	43.32 ± 1.43	−0.098 ± 0.387	–1.740	0.088
	D	43.15 ± 1.45	43.09 ± 1.40	−0.059 ± 0.414	–0.878	0.386
K2 (D)	A	43.78 ± 1.44	43.76 ± 1.43	−0.027 ± 0.261	–0.940	0.350
	B	44.36 ± 1.51	44.19 ± 1.85	−0.176 ± 1.325	–1.052	0.297
	C	44.59 ± 1.58	44.60 ± 1.78	0.009 ± 0.618	0.102	0.920
	D	43.38 ± 1.51	44.29 ± 1.46	−0.086 ± 0.426	–1.237	0.224

AL, axial lengths; ACD, anterior chamber depth; LT, lens thickness; WtW, white-to-white distance; K1, flat corneal power; K2, steep corneal power; D, diopter.

Findings from IOL power calculation are presented in Tables 2, 3. The findings showed that the IOL power calculated using the SRK/T and Haigis formula did not change significantly following pupil dilation in all four groups (*P* > 0.05). In addition, the IOL power calculated using the Barrett Universal II formula did not show significant differences between pre- and post-dilation in Group B, C, and D (*P* > 0.05). Pre- and post-dilation IOL power calculation showed significant differences only in Group A when the Barrett Universal II formula was used (*P* = 0.004).

**TABLE 2:** The mean intraocular lens (IOL) power (D) calculated by IOLMaster 700 before and after pupil dilation.

		Mean ± SD				
				
IOL formula	Group	Pre-dilation	Post-dilation	Difference	*t*	*p*
SRK T	A	11.44 ± 2.36	11.51 ± 2.33	0.065 ± 0.384	1.553	0.124
	B	4.97 ± 2.64	4.95 ± 2.71	−0.016 ± 0.411	–0.306	0.760
	C	−0.29 ± 2.98	−0.17 ± 2.83	0.117 ± 0.761	1.055	0.297
	D	−5.49 ± 3.88	−5.50 ± 3.86	0.131 ± 0.721	–0.112	0.911
Haigis	A	11.70 ± 2.61	11.78 ± 2.57	0.076 ± 0.433	1.628	0.107
	B	5.35 ± 2.58	5.38 ± 2.61	0.032 ± 0.358	0.704	0.484
	C	0.36 ± 2.70	0.47 ± 2.61	0.106 ± 0.707	1.032	0.307
	D	−4.68 ± 3.30	−4.71 ± 3.38	−0.026 ± 0.697	–0.233	0.817
Barrett universal II	A	11.54 ± 2.46	11.65 ± 2.44	0.112 ± 0.348	2.959	0.004
	B	5.56 ± 2.22	5.62 ± 2.26	0.063 ± 0.353	1.426	0.159
	C	1.27 ± 2.35	1.31 ± 2.36	0.043 ± 0.540	0.540	0.592
	D	−2.67 ± 2.31	−2.71 ± 2.33	−0.039 ± 0.456	–0.534	0.597

D, diopter.

**TABLE 3:** The intraocular lens (IOL) power (D) difference before and after pupil dilation as discrete variable with changes in intervals of 0.5D.

IOL formula	Group	0.0D	0.5D	1.0D	1.5D	2.0D	2.5D	3.0D	3.5D	No. of eyes
SRK T	A	55 (64.7%)	27 (31.8%)	2 (2.4%)	0	1 (1.2%)	0	0	0	85
	B	37 (58.7%)	24 (38.1%)	0	2 (3.2%)	0	0	0	0	63
	C	24 (51.1%)	20 (42.6%)	1 (2.1%)	0	0	0	1 (2.1%)	1 (2.1%)	47
	D	14 (36.8%)	17 (44.7%)	6 (15.8%)	0	0	0	1 (2.6%)	0	38
Haigis	A	49 (57.7%)	32 (37.7%)	2 (2.4%)	1 (1.2%)	1 (1.2%)	0	0	0	85
	B	42 (66.7)%	19 (30.2%)	1 (1.6%)	1 (1.6%)	0	0	0	0	63
	C	29 (61.7%)	14 (29.8%)	2 (4.3%)	0	0	0	2 (4.3%)	0	47
	D	16 (42.1%)	16 (42.1%)	5 (13.2%)	0	0	0	1 (2.6%)	0	38
Barrett universal II	A	57 (67.1%)	24 (29.3%)	3 (3.5%)	1 (1.2%)	0	0	0	0	85
	B	42 (66.7%)	19 (30.2%)	1 (1.6%)	1 (1.6%)	0	0	0	0	63
	C	31 (66.0%)	14 (29.8%)	1 (2.1%)	0	0	0	1 (2.1%)	0	47
	D	22 (57.9%)	15 (39.5%)	0	0	1 (2.6%)	0	0	0	38

D, diopter.

However, when IOL power was calculated as a discrete variable with changes in intervals of 0.5D, significant differences were observed between pre- and post-dilation IOL power (Table 3). Calculation using the SRK/T formula showed that dilation changed the IOL power by more than 1.0D in 3 (3.5%), 2 (3.2%), 3 (6.4%), and 7 (18.4%) eyes of Group A, B, C, and D, respectively. Calculation using the Haigis formula showed that dilation changed the IOL power by more than 1.0D in 4 (4.7%), 2 (3.2%), 4 (8.5%), and 6 (15.8%) eyes in Group A, B, C, and D, respectively. Calculation using the Barrett Universal II formula indicated that dilation changed the IOL power by more than 1.0D in 4 (4.7%), 2 (3.2%), 2 (4.3%), and 1 (2.6%) eyes in Group A, B, C, and D, respectively. Notably, IOL power differences incurred by dilation for the three formulas in Group A and B were all ≤2.0D, whereas Group C and D exhibited ≥3.0D differences. Dilation altered the IOL power by 3.0D in 1 eye (2.1%) and 3.5D in 1 eye (2.1%) as calculated using the SRK/T formula, by 3.0D in 2 eyes (4.3%) as calculated using the Haigis formula, and by 3.0D in 1 eye (2.1%) as calculated using the Barrett Universal II formula in Group C. Dilation altered the IOL power by 3.0D in 1 eye (2.6%) as calculated using the SRK/T formula, and by 3.0D in 1 eye (2.6%) as calculated using the Haigis formula in Group D.

## Discussion

High myopia is especially prevalent in East Asia and its prevalence appears to be on the rise. A highly myopic eye is characterized by early-onset nuclear cataract as well as development of a series of choroidal and retinal lesions. Therefore, fundus conditions should be examined carefully before cataract operation with mydriasis in cataract patients with high myopia to reduce the incidence rates of postoperative fundus complications. However, studies have not explored whether pre-operation biometry measurements could be performed with mydriasis on these patients. Previous studies only evaluated the effects of mydriasis on biometry measurements and IOL power calculation for eyes of average AL (2, 4–10). The effects of mydriasis on biometry measurements and IOL power calculation for eyes with high to extremely high myopia (26.0 mm ≤ AL < 36.0 mm) should be explored.

Errors in pre-operative AL measurements are associated with poor refractive outcomes of cataract surgery (11). The majority of studies reported that AL measurements following mydriasis do not result in significant changes (6, 7, 12–16). The results from the present study showed that there was no statistically significant difference between pre- and post-dilation AL of cataract patients with high myopia, which is consistent with previous findings. However, limited number of studies reported contrary findings. Cheng and Hsieh (17) used partial coherence interferometry and reported that AL elongated 13 μm (*p* < 0.001) after application of 0.4% tropicamide in children with a mean age of 9.1 ± 2.8 years. Tuncer et al. (5) conducted a study that comprised 10–20, 30–40, and 50–60 year-old participants. The findings showed AL elongated 10 μm for the 10–20 and 50–60 year-old groups (*p* < 0.05) after application of 1% cyclopentolate, however, no significant change was observed in the 30–40 year-old group. They hypothesized that the lens-ciliary body move posteriorly following cycloplegia, inducing a compression force that pushes toward the vitreous cavity leading to temporary AL elongation. Gao et al. (18) used A-scan and reported that AL markedly increased by 20 μm (*p* < 0.05) in hyperopic eyes in Chinese children with a mean age of 9.6 ± 2.3 years who presented with cycloplegia induced by atropine. Notably, an average AL decreased of 20 μm (*p* < 0.05) was observed in eyes with mild to moderate degrees of myopia after cycloplegia in the same study. Gao et al. (18) hypothesized that accommodation may induce a relative forward movement of the apex of the steep cornea in myopic eyes leading to increase in AL, as myopic eyes often have greater corneal power, therefore, cycloplegia results in decrease in AL. Although these studies reported different findings, the results from these studies are not directly comparable to ours. Participants in previous reports were limited to healthy individuals that did not present with cataracts and high myopia. In addition, two studies only included healthy children as participants.

The dominant theory indicates that the lens becomes flatter and shifts posteriorly when cycloplegia eliminates accommodation, therefore ACD increases and LT decreases (2, 7, 13, 16, 19–24). Previous studies utilized IOLMaster reported that ACD increased significantly by 60–120 μm (*p* < 0.05) whereas LT decreased markedly by 20–100 μm (*p* < 0.05) after cycloplegia in eyes with average AL (22.0–26.0 mm) presenting with or without cataract (2, 15, 16, 19). The present findings showed that the post-dilation mean ACD increased significantly in Group A–D by 90 μm, 94 μm, 116 μm, and 109 μm (*P* = 0.000 in all groups), respectively, whereas post-dilation mean LT decreased significantly by 14 μm, 22 μm, 15 μm, and 18 μm (*P* = 0.000, 0.000, 0.001, and 0.003), respectively, which is consistent with previous findings. However, the lens nucleus in the elderly becomes harder and less compressible and its capsule becomes less elastic compared with that of children and young adults (18, 24). Arriola-Villalobos et al. (3) reported that when ACD significantly deepened (*p* < 0.001) after cycloplegia, there were no marked differences in LT (*P* = 0.847) for cataract patients with a mean age 74.7 ± 7.5 years (range 56–90).

The findings in the current study showed significant changes in WtW after cycloplegia. Post-dilation WtW values were significantly higher in Group A, B, and C by 81 μm, 90 μm, and 60 μm (*P* = 0.001, 0.001, and 0.025), respectively, compared with the pre-dilation WtW values. The post-dilation WtW in Group D also increased by 68 μm relative to the pre-dilation WtW (*P* = 0.053), however, the difference was not significant. Only few studies have explored WtW changes following cycloplegia. Using IOLMaster, studies that comprised healthy eyes with average AL (22.0–26.0 mm) without cataracts reported a 40–100 μm (*p* < 0.05) increase in WtW values following cycloplegia (2, 7, 10, 13, 20). However, these studies did not report whether this increase arose from artifacts or anatomical changes. The IOLMaster determines WtW by obtaining a digital image of the eye and detecting the limbus based on bright-dark contrast between the dark iris and the pale sclera. Pupil dilation with iris bunching increases tissue darkness, thus the device may inaccurately overestimate the limbus to be closer to the sclera (2, 10, 13). In fact, this bright-dark contrast boundary is not clear and any illumination changes may affect the final WtW measurements (25).

Accurate measurement of K is important for IOL power calculation since a 1.00D error in keratometry measurement corresponds to an error of 0.8–1.3D in IOL power (26). Some studies reported insignificant change in K values following cycloplegia (3, 6, 13, 19), whereas other studies reported a significant decrease in K values (5, 17, 18). Notably, participants in most studies (3, 6, 19) that did not report any changes in K values were cataract patients. Similar to these studies, we did not find significant differences between pre- and post-dilation K1 and K2 values in cataract patients with high myopia (*P* > 0.05). On the contrary, participants in the studies which reported decreased K values following cycloplegia were healthy children and adults without cataracts (5, 17, 18). Justified the eyeballs of healthy children and young adults as “elastic containers,” they hypothesized that flexibility of the outer coat of the eyeball in children and young adults is better compared to that of the elderly, thus when cycloplegia eliminates the contractive force of the ciliary muscle, the centripetal force on the sclera spur reduces, causing corneal flattening (17, 18). Moreover, the device used in the present study was the IOLMaster. Shammas et al. (27) reported that reproducibility of K readings using IOLMaster was relatively poor in steep corneas (*K* > 42.0D) with higher fluctuations. In the current study, a mean *K* > 42.0D was recorded in 179 eyes (76.82%). Further studies should validate these findings using corneal topography devices, considering the relatively limited reliability of the IOLMaster as a keratometer.

The SRK/T formula has previously been suggested as the most accurate formula for eyes with long AL (>27.0 mm) (28). Recently, studies have reported that formulas including Haigis, Barrett Universal II, and Olsen are more accurate for long eyes (AL ≥ 26.0 mm) compared to the SRK/T formula (29–32). However, agreement has not been reached. Some studies investigated the accuracy of different formulas for IOL power calculation of long eyes and the findings did not reveal significant differences in accuracy among different formulas (33–36). The optimal formula for IOL power calculation following cycloplegia has not been established. Studies using SRK/T and Haigis formulas in eyes with average AL (22.0–26.0 mm) reported that cycloplegia did not statistically affect the IOL power calculation results (3, 6, 12). Our results on eyes with high myopia were consistent with previous findings that the SRK/T and Haigis formulas showed no statistically significant differences between pre- and post-dilation IOL power in the four groups. A statistically significant increase of 0.112D (*P* = 0.004) was observed in Group A after cycloplegia using the Barrett Universal II formula. This statistical difference has very limited clinical significance since a 0.1D error in IOL power corresponds to an error of approximately 0.067D in the spectacle plane.

In actual practice the IOLs are in steps of 0.5D. Notably, analysis of the optimal IOL selected for emmetropia between the two different pupil conditions of each eye showed very different results. Calculated with the three different formulas for Group A–D showed that only 36.8–67.1% of eyes did not change IOL following dilation, whereas 2.6–18.4% of eyes showed IOL changes ≥1.0D post-dilation. Similar results were obtained for eyes with average AL in previous studies. For instance, Adler et al. (6) reported that although the difference in mean IOL power was not significant, when IOL power was calculated as discrete variable, only 66.4% of eyes did not change IOL with the SRK/T formula following pupil dilation. A 1.0D difference in IOL corresponds to approximately 0.72D in the spectacle, therefore, this difference could significantly affect the refractive outcome of the cataract surgery. Moreover, the magnitude of the pre- and post-dilation difference was seemingly positively correlated with AL. IOL power differences associated with dilation for the three formulas were ≤2.0D for Group A and B, whereas Group C and D exhibited ≥3.0D differences. Group D had the highest percentage of eyes with ≥1.0D pre- and post-dilation IOL power differences (18.4% for the SRK/T formula and 15.8% for the Haigis formula). Notably, Barrett Universal II formula had the best concordance between different pupil conditions.

The current study has several limitations. First, posterior staphyloma was present in about 1/3 to 1/2 of eyes with high myopia. A previous study reported a significant correlation between posterior staphyloma classification and refractive error after cataract surgery (37). In the current study eyes with posterior staphyloma were not excluded. Moreover, relevant fundus examination was not performed to classify the types of posterior staphyloma. Therefore, further studies should be conducted to explore the effect of posterior staphyloma on ocular parameter measurement and IOL power prediction following pupil dilation. Second, the age of our study subjects ranged from 29 to 84 years old. Previous findings indicated that ocular morphologic changes and loss of accommodation are inevitable consequences of aging. It is possible that the eyes response to cycloplegic agents differently at different ages. Thus, further studies should be conducted to explore the effect of aging on the results of the current study. Third, where pupil dilation without light reflex commonly considered as a hallmark for cycloplegia, the concept of mydriasis and cycloplegia is different. Tropicamide is widely used in clinical practice, however, it is not the gold standard agent for cycloplegia. Measurement bias may have been induced in the current study since the ciliary muscle may not have been fully relaxed following 45 min application of tropicamide. However, this reflects the real biometric measurements and corresponding IOL power calculation results in daily clinical practice. Lastly, the inclusion of data from both eyes of some subjects in the study may have had a coupling effect in the statistical analysis.

To the best of our knowledge, previous studies have not reported changes in ocular parameters following pupil dilation in cataract patients with high myopia. The present findings indicate that pupil dilation with tropicamide in long eyes does not cause significant changes in AL and K values. However, it causes significant increase in ACD and WtW values and significant decrease in LT value. Although there was no statistically significant effect of pupil dilation on the corresponding mean IOL power as calculated using the SRK/T and Haigis formulas, surgeons should be careful about the effect of pupil dilation on IOL power prediction as extreme cases were observed in the current study for these two formulas. The Barrett Universal II formula is more promising for IOL power calculation for different pupil conditions in long eye. However, this finding should be further verified through sufficiently sized trials.

## Data availability statement

The raw data supporting the conclusions of this article will be made available by the authors, without undue reservation.

## Ethics statement

The studies involving human participants were reviewed and approved by Shenzhen Eye Hospital. The patients/participants provided their written informed consent to participate in this study.

## Author contributions

WX, MY, KZ, and SC contributed to study design, data acquisition, data analysis, and manuscript preparation. JW, YW, YQ, and XH contributed to data acquisition and data analysis. XL, NF, and SL contributed to study conception and manuscript modification. All authors contributed to the article and approved the submitted version.
